# The Medical Profession in the United States

**Published:** 1846-04

**Authors:** 


### THE MEDICAL PROFESSION IN THE UNITED STATES.

The condition of the medical profession in this country, and the causes which tend to impede its progress, are topics very ably discussed by Dr. Stewart, of New York, in a pamphlet of 30 pages which we have read with deep interest. We cannot say we read it with pleasure, for a contemplation of the evils which beset the profession is calculated to inspire very different emotions. Still, it becomes us to look the evils in the face, and with a resolute spirit set about their correction. "Old sores," it has been said, "need vinegar as well as oil," and in that view of the case, Dr. Stewart has applied the corrosive. He compares, in the subjoined passage, the system of medical education in Europe with that which prevails in our country:

"All the more important subjects, and especially practical anatomy, and clinical medicine and surgery, are there thoroughly taught. The student is not only required to dissect, but is examined on Dissection, whereas, here a very irregular attendance on the dissecting-room, probably during a few evenings only in each session, is all that is expected of our pupils, and indeed, in some cases, they are notified publicly, beforehand, that though advised to do so, they will not be required to dissect in reality. Little or no attention to hospital practice is required from the student here. Some of our colleges exact from him that he shall purchase a ticket of admission to a hospital, when one is convenient, but there the matter rests. And this even is not always required; and if not obligatory on them to do so, how can it be expected that students, when they have so much else to attend to, will go to the expense of procuring a ticket, or after getting it, will take the trouble to attend the practice of these institutions? That they do not do so, is I think, very evident, from the fact that out of upwards of six hundred in attendance at the New York Colleges during the present session, only about one in eight have applied for the privilege of visiting our City Hospital! And yet this is known to be the only one here to which they can obtain access. The all important branch of clinical instruction then is not taught to students here, at all events in a satisfactory manner; for the cliniques attached to the schools in this city, in Philadelphia, and elsewhere, though useful, can never present to them the advantages that they would derive from examining patients, and following their treatment, in a regular and well organized hospital, and under the direction of qualified teachers."

Having exhibited the profession as it is, Dr. S. enumerates the causes which have brought it to its present condition, and are still opposed to its improvement. We would gladly copy from the discourse largely if we had space. Its ethics are of the purest order, and its admonitions and suggestions worthy of the serious regard of all American physicians.

